# Supplementary material for: DMRforPairs: identifying Differentially Methylated Regions between unique samples using array based methylation profiles
Source: BMC Bioinformatics. 2014 May 15;15:141. doi: 10.1186/1471-2105-15-141 (PMC4046028; doi:10.1186/1471-2105-15-141)
Supplement: Additional file 2 — DMRforPairs output for the comparison of A431-MCF7 and NA17018-NA17105. Please start from the HTML files in each folder. Available via the BMC Bioinformatics website. [file 1471-2105-15-141-S2.zip › 1394847754114233_MOESM2_ESM/A431_MCF7/figures/34555.pdf]

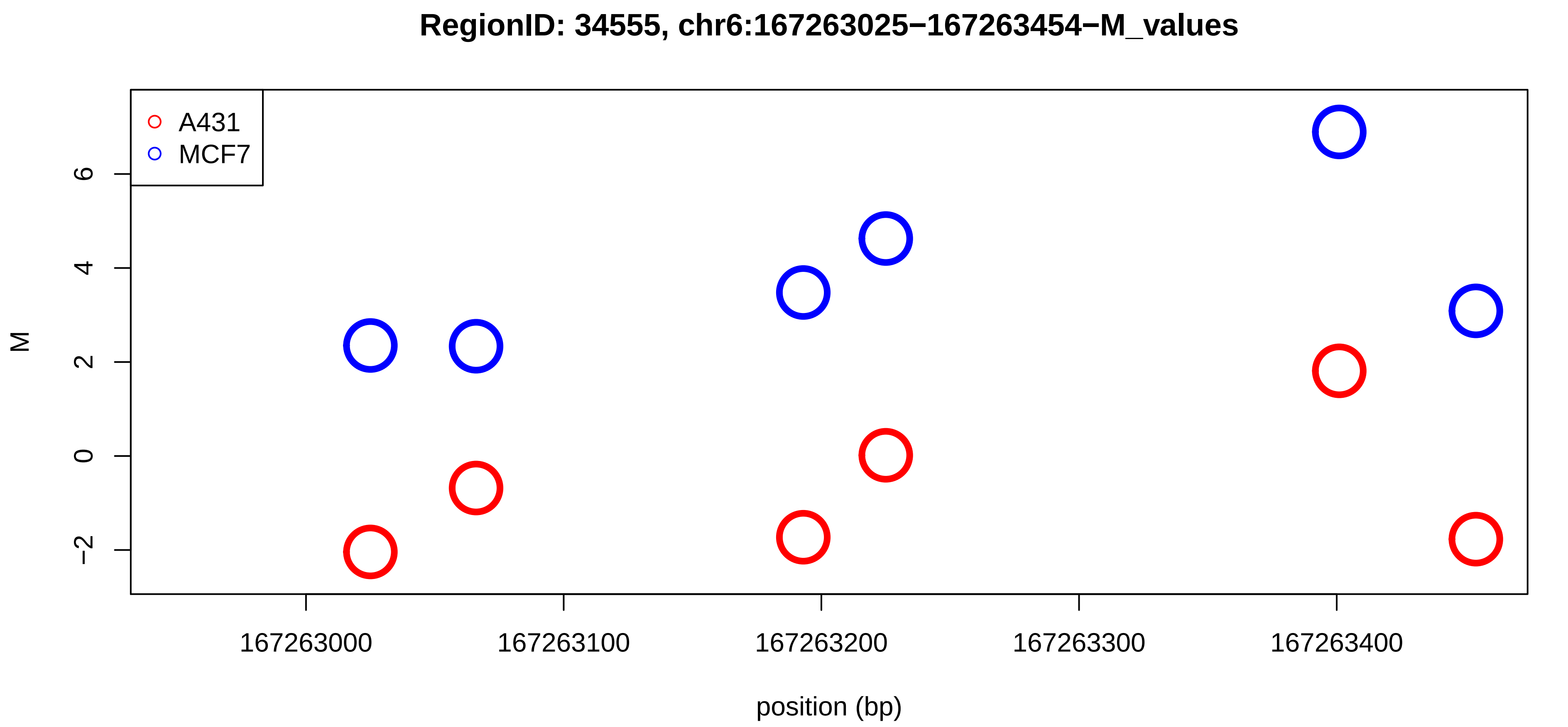

RegionID: 34555, chr6:167263025−167263454−M_values
A431
MCF7
M
−2
0
2
4
6
167263000
167263100
167263200
167263300
167263400
position (bp)

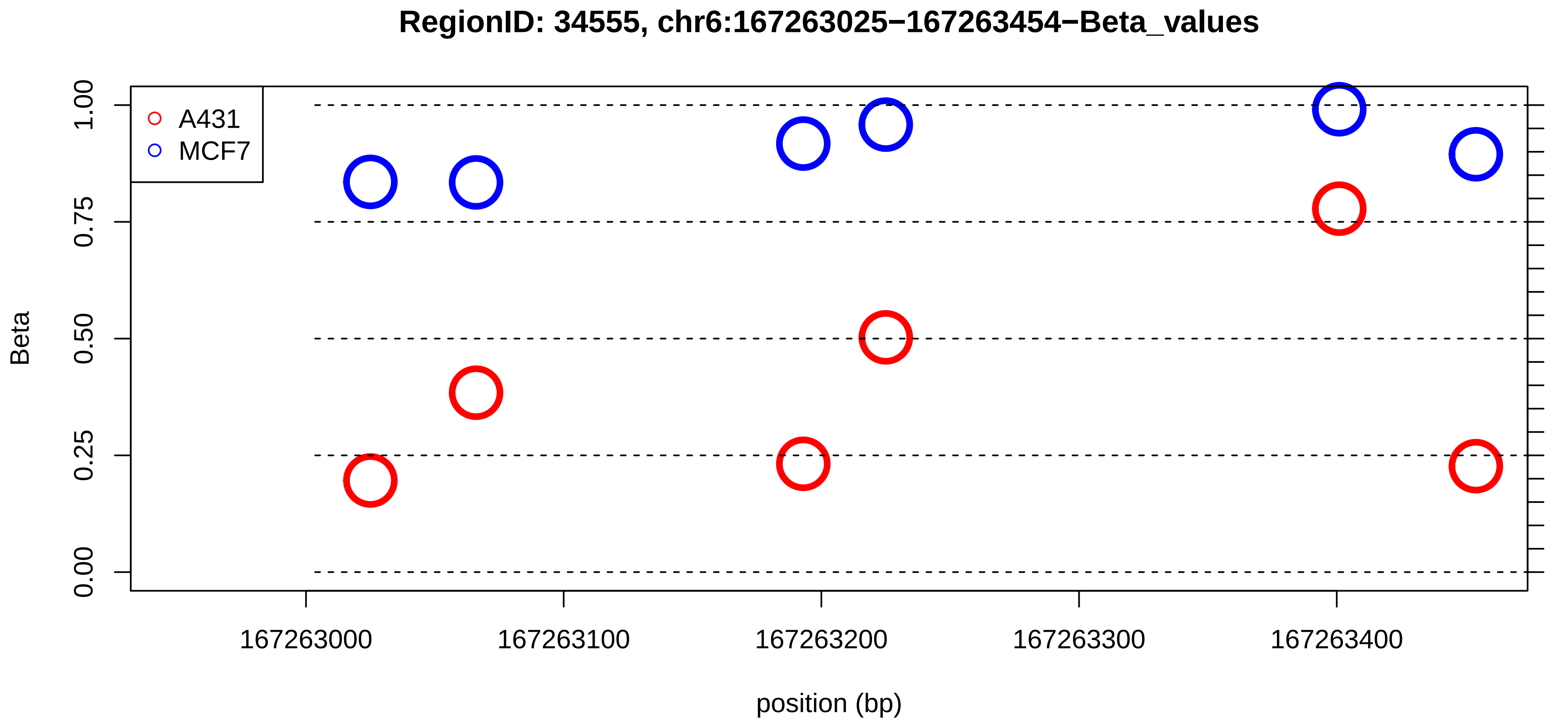

RegionID: 34555, chr6:167263025−167263454−Beta_values
A431
MCF7
Beta
0.00
0.25
0.50
0.75
1.00
167263000
167263100
167263200
167263300
167263400
position (bp)

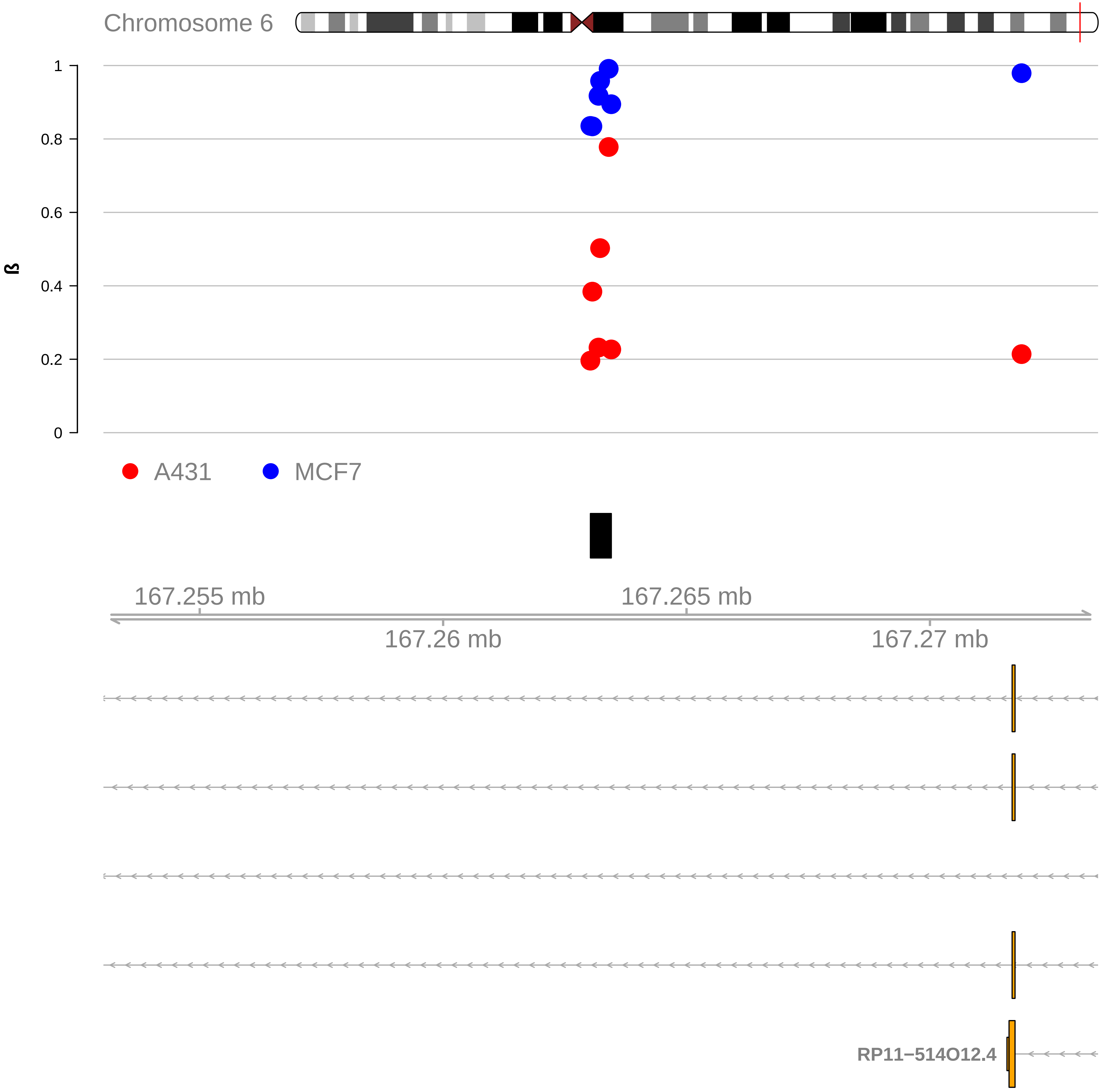
Chromosome 6
ß
1
0.8
0.6
0.4
0.2
0
A431
MCF7
167.255 mb
167.265 mb
167.26 mb
167.27 mb
RP11−514O12.4
